# Chemical Landscape of Adipocytes Derived from 3T3-L1 Cells Investigated by Fourier Transform Infrared and Raman Spectroscopies

**DOI:** 10.3390/ijms252212274

**Published:** 2024-11-15

**Authors:** Karolina Augustyniak, Monika Lesniak, Maciej P. Golan, Hubert Latka, Katarzyna Wojtan, Robert Zdanowski, Jacek Z. Kubiak, Kamilla Malek

**Affiliations:** 1 Faculty of Chemistry, Jagiellonian University, Gronostajowa 2, 30-387 Krakow, Poland; karolina.augustyniak@doctoral.uj.edu.pl (K.A.); hubert.latka99@gmail.com (H.L.); k.wojtan@student.uj.edu.pl (K.W.); 2 Doctoral School of Exact and Natural Sciences, Jagiellonian University, Prof. Stanislawa Lojasiewicza 11, 30-348 Krakow, Poland; 3 Laboratory of Molecular Oncology and Innovative Therapies, Military Institute of Medicine—National Research Institute, Szaserow 128, 04-141 Warsaw, Poland; mlesniak@wim.mil.pl (M.L.); mgolan@aps.edu.pl (M.P.G.); rzdanowski@wim.mil.pl (R.Z.); 4 Institute of Psychology, The Maria Grzegorzewska University, Szczesliwicka 40, 02-353 Warsaw, Poland; 5 Dynamics and Mechanics of Epithelia Group, Institute of Genetics and Development of Rennes (IGDR), French National Centre for Scientific Research (CNRS), Faculty of Medicine, University of Rennes, UMR 6290, 35043 Rennes, France

**Keywords:** adipocytes, cellular compartments, FTIR and Raman spectroscopy imaging, histochemical staining

## Abstract

Adipocytes derived from 3T3-L1 cells are a gold standard for analyses of adipogenesis processes and the metabolism of fat cells. A widely used histological and immunohistochemical staining and mass spectrometry lipidomics are mainly aimed for examining lipid droplets (LDs). Visualizing other cellular compartments contributing to the cellular machinery requires additional cell culturing for multiple labeling. Here, we present the localization of the intracellular structure of the 3T3-L1-derived adipocytes utilizing vibrational spectromicroscopy, which simultaneously illustrates the cellular compartments and provides chemical composition without extensive sample preparation and in the naïve state. Both vibrational spectra (FTIR—Fourier transform infrared and RS—Raman scattering spectroscopy) extended the gathered chemical information. We proved that both IR and RS spectra provide distinct chemical information about lipid content and their structure. Despite the expected presence of triacylglycerols and cholesteryl esters in lipid droplets, we also estimated the length and unsaturation degree of the fatty acid acyl chains that were congruent with known MS lipidomics of these cells. In addition, the clustering of spectral images revealed that the direct surroundings around LDs attributed to lipid-associated proteins and a high abundance of mitochondria. Finally, by using quantified markers of biomolecules, we showed that the fixative agents, paraformaldehyde and glutaraldehyde, affected the cellular compartment differently. We concluded that PFA preserves LDs better, while GA fixation is better for cytochromes and unsaturated lipid analysis. The proposed analysis of the spectral images constitutes a complementary tool for investigations into the structural and molecular features of fat cells.

## 1. Introduction

Among all eukaryotic cells, adipocytes exhibit a unique architecture. Besides the typical cellular structures, like the nucleus, endoplasmic reticulum (ER), and mitochondria, fat cells feature an abundance of lipid droplets (LDs). As adipocytes’ primary cellular organelles, LDs encompass greater than 95% of the entire cell body. These spherical structures can reach up to 100 µm in diameter and are made up of a neutral lipid core, packed mainly with cholesteryl esters (CEs) and triacylglycerols (TAGs), which is surrounded by a phospholipid monolayer membrane [1,2].

Due to the high content of LDs, adipocytes were initially associated with energy storage. However, it is now clear that fat cells have additional roles connected with the presence of lipid-droplet-binding proteins and interactions between LDs and other organelles, e.g., endoplasmic reticulum (ER), mitochondria, or plasma membrane [3]. As such, adipocytes appear to be fully involved in cell signaling, lipid metabolism, and inflammation processes [4]. On the other hand, overly excessive lipid accumulation is crucial to the pathophysiology of common metabolic disorders, such as obesity [5], diabetes [6], and atherosclerosis [7,8]. Due to the rapid increase in the prevalence of these diseases in the current human population, an interest in studying adipocyte cells has grown within the last decades. It is, thus, critical to develop novel and adequate methodologies for studying the morpho-chemistry of adipocytes and their impact on the above-listed pathologies [9].

Currently, to facilitate the fat cell examination with microscopic techniques, chemical dyes (e.g., osmium tetroxide, Oil Red O—ORO) and fluorescent probes (BODIPY, LipidTOX, Nile Red), which target lipidic species only, are used [10]. On the other hand, immunofluorescence labeling (IF) is employed to reveal lipid-droplet-associated protein distribution [11]. Fluorescence and optical microscopies can identify lipid accumulations but lack the quantitative information to describe their chemism. Also, with the abovementioned staining methods, an appropriate cell fixation is obligatory to ensure the accurate visualization of fat cell compartments. Because phospholipid membranes are not resistant to drying or treating with alcohol-based reagents, adipocytes remain very vulnerable to fixation and staining [12]. For instance, paraformaldehyde (PFA) preserves the structure of lipid droplets more efficiently than cold ethanol and acetone because PFA does not cause LD fusion [9]. On the other hand, to detect LD-related proteins with IF labelling, glutaraldehyde (GA) and its mixtures must be tested for their correct identification [13]. Therefore, a complex sample preparation protocol is required to preserve cellular structures within adipocytes and efficiently perform immunostainings.

Although imaging tools provide extensive information about fat cell morphology, they only allow for the limited determination of their biochemical composition. As a remedy for this, lipidomics has arisen as a fundamental technique for studying lipid chemical compositions. This mass-spectrometry-based (MS) technique provides precise information for identifying and quantifying lipid species. The alternations in the examined systems can be determined by analyzing their structures, interactions, and dynamics [14]. However, to prepare the sample, all lipids must be extracted first. This disrupts the structure of complex systems, such as cell organelles, making it impossible to observe their naïve form [15]. Furthermore, the results must be interpreted as a whole without being attributed to cellular compartments, which limits insight into subcellular chemistry. Additionally, a minimum of 1 × 10^6^ cells is required [16].

The alternative microscopic techniques are Raman (RS) and Fourier transform infrared (FTIR) spectroscopies and their imaging modalities. They detect vibrational bands to the lipid chemistry in the whole cells and their compartments, i.e., the C=C, C-C bonds (RS), the CH_2_, and CH_3_ bonds in the fatty acid acyl chains (RS/FTIR), as well as the carbonyl C=O group in fatty acids and their triacylglycerols (FTIR). The imaging systems operate in two ways, i.e., hyperspectral mapping collecting full-region spectra from a point grid and single-frequency mapping based on determined earlier spectral predictor (e.g., for the CH_2_ band). Both methods are also label-free and non-destructive for cells. So far, single-frequency RS imaging has been most frequently employed to study the spatiotemporal/chemical profiling of adipocytes revealing, i.e., the lipid droplet-dependent composition changes to various culture conditions in human adipose-derived stem cells, similarities in lipidic species in myocellular LDs of individuals with type 2 diabetes, as well as heterogeneous lipid uptake, which was droplet-size-dependent and time-dependent [17,18,19,20]. This fast sample mapping mainly provides the visualization of LDs and their abundance and size (>0.2 µm) [21,22]. A report has also shown the distribution of the entire pool of proteins in fat cells [23]. Hyperspectral Raman imaging has also been applied to monitor the unsaturation degree of LD fatty acids and the presence of esterified fatty acids to understand stimuli effects mimicking metabolic disorders within adipocytes [22,24]. In turn, the corresponding FTIR imaging has been successfully used to predict adipose-derived mesenchymal stem cells’ adipogenesis and action on antiadipogenic agents. It has been proposed as a cell-sorting technique [25,26,27,28]. Recently, single-frequency imaging incorporated in optical photothermal infrared microscopy (OPTIR) has been combined with the isotopic labeling of glucose to track de novo lipogenesis in 3T3-L1 adipocytes by showing the distribution of esterified lipids and proteins [29].

Surprisingly, the comprehensive chemical information encoded in the FTIR and RS spectra of fat cells has not yet been exploited, particularly regarding their complementarity. RS spectrum is not only specific to the unsaturation degree of lipids but also to phospholipids (PLs), bases of nucleic acids, aromatic amino acids, and hemoproteins, while FTIR spectrum reveals the presence of triacylglycerols, cholesterol esters, and changes in the length of FA chains and the secondary structures of proteins [25,30]. Considering the capacity of FTIR and Raman spectroscopy imaging to evaluate the cellular morpho-chemistry promptly and in a label-free way, we report the full characteristics of fat cells and their cellular microenvironment in this work. As the model, we chose 3T3-L1-derived mature adipocytes, because they are an important in vitro model of white adipocytes. 3T3-L1 cells have been commonly used to investigate the underlying molecular processes of adipogenesis and to assess the potential utility of various substances and nutrients in obesity treatment [31]. We report multiple IR and RS qualitative and quantitative spectral parameters, showing the entire metabolic machinery of the adipocyte. We also referred to often-used optical microscopy, histological, and immunochemical staining. In addition, we studied whether fixative agents affect the spectral markers, and thus, if specific cell preservation is required for spectroscopic imaging. The experiments reported here led us to hypothesize that applying FTIR and RS microscopy, together or separately, can monitor various lipid- and protein-related processes in adipocytes commonly visualized by time- and material-consuming bioassays. To our knowledge, there is no study showing a detailed description of the chemical composition of these cells related to their intracellular morphology obtained by label-free molecular imaging. The established IR and Raman markers and their combination was the focus of that study and is discussed here in terms of their key role in the function of the adipocyte.

## 2. Results

### 2.1. Optical Microscopy and Staining for Adipocyte Profiling

**Figure 1:**
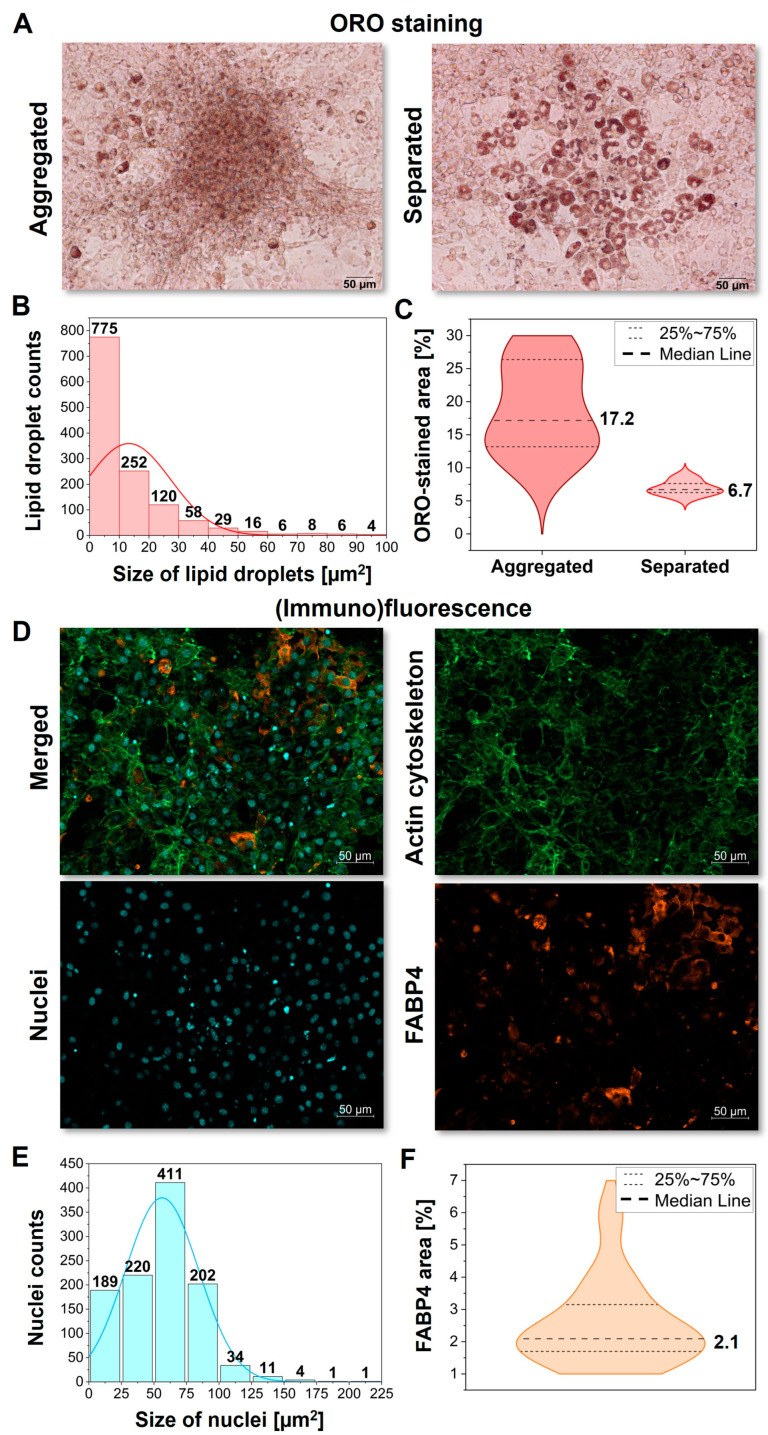
(**A**) Oil Red O (ORO) staining of aggregated (left) and separated (right) adipocytes derived from the 3T3-L1 cells with (**B**) LD size distribution (N = 10 images) and (**C**) a calculated stained area of both types of cells (N = 10 images). (**D**) Fluorescence images (N = 12 images) revealing the actin cytoskeleton (green), nuclei (blue), and immunolocalization of FABP4 protein (orange) of mature adipocytes with a calculated distribution of nuclei size (**E**) and area covered by FABP4 protein (**F**). The cells were fixed with 4% paraformaldehyde.

The images of Oil Red O staining were collected first to exhibit the morphology of lipidic occlusions formed within the mature adipocytes, determine their size, and at the same time, confirm their lipidic character (Figure 1A–C). As ORO binds hydrophobic and neutral lipids, such as triglycerides, diacylglycerols, and cholesterol esters present in the interior of the lipid body, it is the most often used tool for the in situ screening of fat cells as well as adipose tissue. In addition, fluorescence microscopy was applied to visualize the actin cytoskeleton, nuclei, and the fatty-acid-binding protein 4 (FABP4), also known as adipocyte protein 2 (aP2) detected by immunofluorescence (Figure 1E). The bright-field optical images of 3T3-L1-derived adipocytes are additionally included in the Supplementary Materials (Figure S1).

The presented examples of ORO images exhibit the diversity of lipid bodies’ numerosity and size between single mature adipocytes (Figure 1A). Here, we examined ca. 1200 LDs and established the distribution profile of their size. The obtained histogram showed the left-side skewed distribution of this parameter with a maximum at ca. 13 µm^2^ for a single LD (Figure 1B). Most lipid droplets are smaller than 10 µm^2^, but some can reach up to 100 µm^2^. Such heterogeneity of their formation underlines a need for imaging tools able to detect them. Furthermore, the collected images showed two types of differentiated 3T3-L1 cells, according to the lipid accumulation. In the first case, the cells were highly aggregated, forming a homogenous lipidic film due to the fusion of small LDs (Figure 1A, left). On the other hand, the separated and well-developed adipocytes were observed (Figure 1A, right). The statistical analysis of the ORO-stained area for both cases exhibited completely diverse results, with a median of 17.2% and 6.7% for aggregated and separated adipocytes, respectively (Figure 1C).

The immunohistochemical staining of FABP4 and the fluorescence staining of actin and nuclei, in turn, illustrate the subcellular composition of the adipocytes. The actin cytoskeleton is a highly dynamic structure essential to maintaining cellular shape and providing the evenly distributed structural support of fibers in the cells (Figure 1D, green). The nuclei of mature adipocytes are oval-shaped with low-condensed chromatin (Figure 1D, blue). The histogram showing their size distribution demonstrates a normal distribution among ca. 1000 examined nuclei with an average size from 50 to 75 µm^2^ typical for the mature adipocytes (Figure 1E) [32]. In turn, FABP4 is not evenly distributed, as some cells have a significantly higher concentration of this protein in the cytoplasm (Figure 1D, orange). FABP4 directly regulates the size of lipid droplets, and its higher concentration is correlated with the completion of the adipogenesis process, which may differ between the cells. The statistical analysis of the collected IF images revealed that, on average, 2.1% of their area is covered by the FABP4 signal (Figure 1F).

Even though the ORO and IF microphotographs (Figure 1A,D) provided insight into the adipocyte morphology and confirmed the lipid accumulation, with this analysis alone, the chemical composition of the subcellular compartments of adipocytes remains unknown.

### 2.2. Morphological and Chemical Description of Adipocytes by Raman and IR Markers

**Figure 2:**
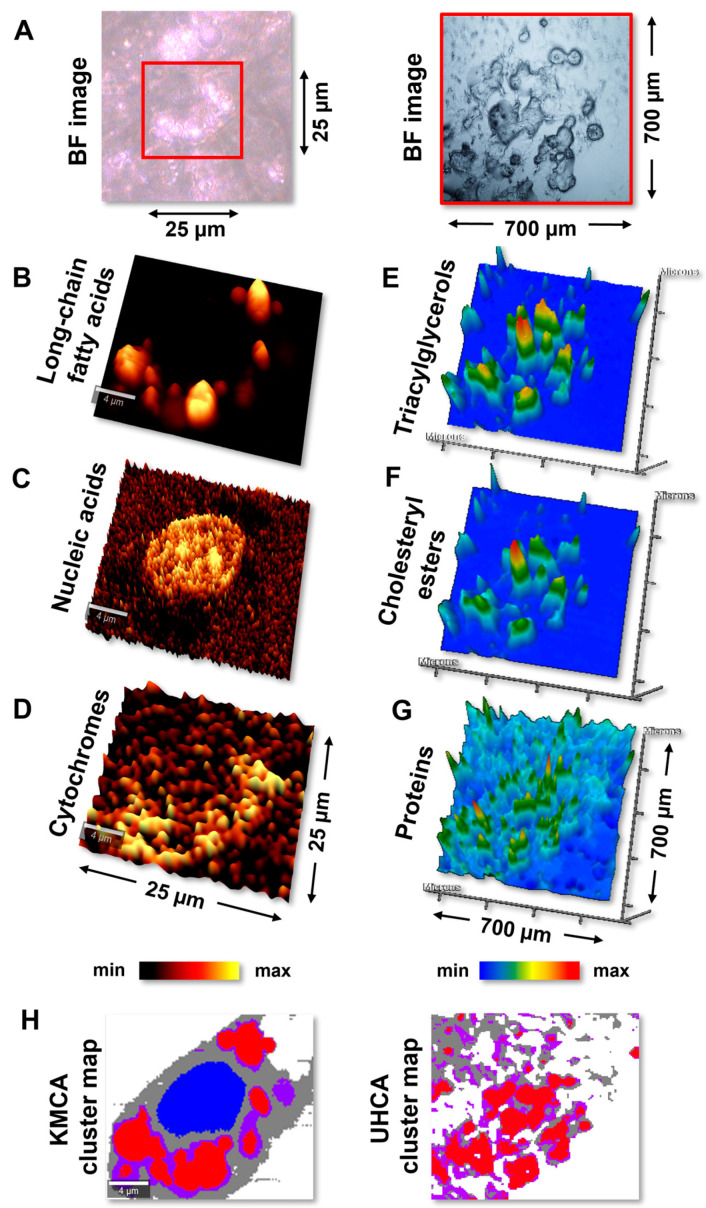
(**A**) Exemplary bright-field images of 3T3-L1-derived adipocytes obtained using Raman (left) and FTIR (right) microscopes. The high-resolution RS microscope enables the chemical imaging of the single cells (ca. 25 µm × 25 µm); whereas, FTIR microscopy depicts several adipocytes within an area of ca. 700 µm × 700 µm. Red squares illustrate the imaged regions. Obtained Raman (**B**–**D**) and FTIR (**E**–**G**) chemical images complementarily depict the distribution of primary components of adipocytes: (**B**) long-chain fatty acids (RS: 2853 cm^−1^), (**C**) nucleic acids (RS: 790 cm^−1^), (**D**) cytochromes (RS: 756 cm^−1^), (**E**) triacylglycerols (IR: 1744 cm^−1^), (**F**) cholesteryl esters (IR: 1169 cm^−1^), and (**G**) proteins (IR: 1651 cm^−1^). The maps were generated using the integral intensities of the marker IR and RS bands. (**H**) Through the similarity-based grouping of RS and IR spectra, the simplified cluster maps were obtained. The corresponding false-color KMCA (left) and UHCA maps (right) of RS and FTIR images, respectively, revealed the presence of the main subcellular compartments of adipocytes, i.e., lipid droplets (red), perilipidic area (purple), nucleus (blue), and cytoplasm (gray). The cells shown here were fixed with 4% PFA.

Maps of the distribution of biocomponents constructed from RS and IR images provided an alternative illustration of the cellular morphology (Figure 2). In the spectroscopic study, we investigated the cells fixed with paraformaldehyde (PFA) and glutaraldehyde (GA). Here, to directly compare the histological and RS/IR images, we present and discuss the data for PFA-fixed cells below.

The high resolution of the RS microscope provided the chemical imaging of the single cells (ca. 25 µm × 25 µm) (Figure 2A, left). In contrast, FTIR microscopy simultaneously depicted several adipocytes within ca. 700 µm × 700 µm (Figure 2A, right). The high-resolution RS maps of the differentiated 3T3-L1 cells revealed the presence of numerous well-separated lipid droplets containing long-chain fatty acids (FAs) (Figure 2B). The LD size varied between 1 and 6 µm^2^, like in the ORO images (Figure 1A,B). In the central part of the cell, an oval-shaped nucleus with an average area of ca. 50 µm^2^ was observed, like the IF stains with bisBenzamide H 33342 trihydrochloride (see Figure 1D,E and Figure 2C). The LDs and the nucleus were surrounded by multiple mitochondria detected by the Raman signal of cytochromes (Figure 2D). The high abundance of mitochondria is essential for highly energetic and continual metabolic changes within adipocytes.

Despite the air-drying process required for FTIR technique, the clusters of adipocytes with visible lipid bodies were still observed (Figure 2A, right). Thus, even though FTIR imaging is characterized by a lower spatial resolution than Raman imaging (ca. 6.1 µm at 1744 cm^−1^ vs. 0.5 µm, respectively), the distribution of the main components of the LD interior, triacylglycerols, and cholesteryl esters was easily detected, and their distribution maps showed their colocalization (Figure 2E,F). The LD-covered area of the cell population constituted ca. 24% of the IR maps, like the ORO stains (Figure 1C). In turn, the distribution of proteins showed their accumulations near the border of the lipid droplets (Figure 2G, red pixels). We assumed that it could be correlated with LD-related proteins (e.g., FABP4) (Figure 1F). As the 1650 cm^−1^ band in the IR spectrum represents all cellular proteins, the less intense, protein-based signal in the map (Figure 2G, light-blue pixels) can be assigned to the actin cytoskeleton (Figure 1D and Figure 2G).

### 2.3. IRRS Spectroscopic-Based Omics

Apart from the spatial information about the adipocyte cellular structure, Raman and FTIR spectra contain encoded biochemical information combining the abilities of omics techniques and IF/histochemical staining. To facilitate the spectral interpretation, we employed dataset reduction methods, such as the cluster analysis of hyperspectral images. Here, *k*-means (KMCA) and unsupervised hierarchical cluster analysis (UHCA) were used for the analysis of RS and FTIR chemical images, respectively (Figure 2H). The clustering algorithms for the RS and FTIR datasets were assessed based on segmentation results, and a distinct strategy was chosen for both imaging modalities according to our previous works [30,33].

**Figure 3:**
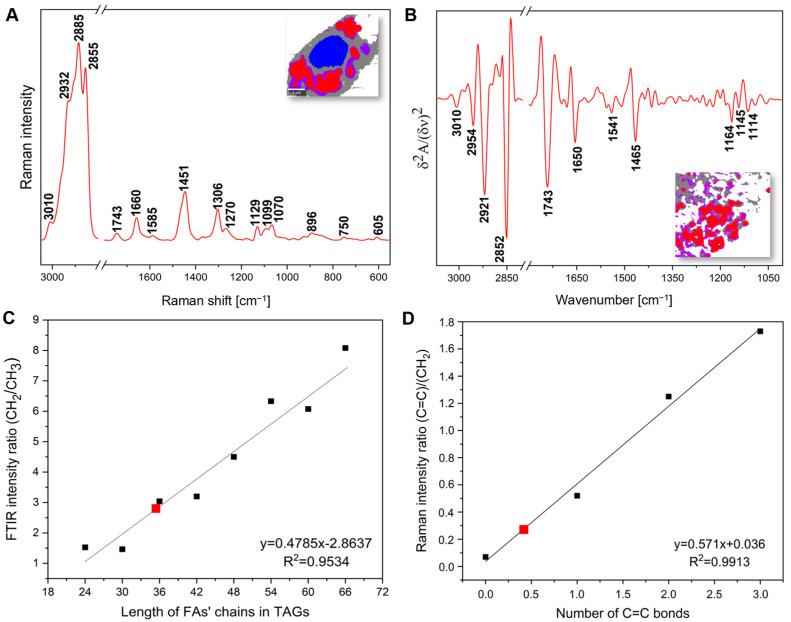
(**A**) The averaged Raman (N = 100) and (**B**) second derivative of FTIR spectra (N = 30) of the lipid droplets from PFA-fixed adipocytes in the spectral regions of 3050–550 cm^−1^ and 3070–1005 cm^−1^, respectively. (**C**) A calibration plot of the length of the FA acyl chains in saturated TAGs determined from the FTIR spectra of TCY—tricaprylin (24:0), TCI—tricaprin (30:0), TLU—trilaurin (36:0), TMA—trimyristin (42:0), TPA—tripalmitin (48:0), TSA—tristearin (54:0), TAR—triarachidin (60:0), and TBH—tribehenin (66:0) based on the ratio of the 2852 and 2954 cm^−1^ bands. A red square marks the ratio for the 3T3-L1 lipid droplets. (**D**) A calibration plot of the unsaturation degree of the FA acyl chain determined from the Raman spectra of SA—stearic acid (18:0), OA—oleic acid (18:1), LA—linoleic acid (18:2), and ALA—α-linolenic acid (18:3) based on the ratio of the 1660 and 1451 cm^−1^ bands. A red square marks the ratio for the 3T3-L1 lipid droplets.

As a result, four classes of different profiles were derived and assigned to lipid droplets, the perilipidic area (PA), cytoplasm, and the nucleus. Due to the low spatial resolution of IR imaging, the nucleus profile was hidden among the LDs. The average RS and the second derivative of FTIR spectra of lipid droplets are displayed in Figure 3A,B, while the spectral profile of the other compartments is summarized in Figure S2 in Supplementary Materials.

Both Raman and FTIR spectra of lipid droplets showed a typical vibrational signature of lipids with the intense high-wavenumber region (3015–2850 cm^−1^) assigned to =CH, -CH_2_, and -CH_3_ functional groups (Table 1). The Raman bands of FAs were observed at 2855, 1451, and 1070 cm^−1^. Saturated fatty acids (SFAs, mainly stearic acid 18:0) and phospholipids (phosphatidylglycerol 40:7, cardiolipin 72:3;5, and sphingomyelin 44:4;2) identified in the previous lipidomic study on 3T3-L1 cells showed their presence through RS bands at 896, 1129, and 1099, respectively [34]. The high content of CEs (RS: 605 cm^−1^; IR: 1178 cm^−1^) and TAGs (RS; IR: 1743 cm^−1^) in the interior of lipid droplets was also notified. According to an MS/MS analysis of LDs in the differentiated 3T3-L1 cells, the dominant classes of lipids contain the ester groups and TAGs (48:2, 48:1, and 48:3), CE (14:1), and monoalkyldiacylglycerides (36:0 and 38:0) [35]. To estimate the length of the FA acyl chains in studied cells, we determined a calibration curve using the CH_2_ and CH_3_ stretching modes in a series of saturated TAGs only, since the contribution of the olefinic groups to this estimate is negligible (Figure 3C). The relationship between the ratio of absorbances assigned to CH_2_ and CH_3_ groups and the length of the acyl chain is linear. Here, this ratio determined from the IR spectrum of the 3T3-L1-derived adipocytes corresponds to trilaurin (36:0), which is the most abundant TAG in LDs.

Unsaturated fatty acids (UFAs) also exhibit their Raman and IR signatures due to the vibrations of the HC=C moiety (RS: 3010, 1660, 1306, and 1270 cm^−1^; IR: 3010 cm^−1^). In addition, the averaged degree of unsaturation of the acyl chain in fatty acids can be estimated by an intensity ratio of the bands assigned to modes of the C=C and CH_2_ groups (RS: 1660/1451 cm^−1^) (Figure 3D). Here, the average number of the C=C bonds was 0.42, implicating that the LD composition is dominated by saturated fatty acids with a low content of mono- and polyunsaturated acyl chains.

Besides the LD class, a cluster analysis of IR and RS images indicated the presence of a thin layer coating lipid droplets (Figure 2H, purple class, the perilipidic area). We encoded it as the perilipidic area (PA). The RS and IR profiles of the PA exhibited that this LD coating was lipid-rich, comparable to the LD composition, but with an increased content of phospholipids specific for the bilayer membranes (RS: 1129 and 1099 cm^−1^; IR: 1340 and 1240 cm^−1^) (Figure 3 and Figure S2, Table 1). Notably, the high content of lipids along with the Raman signals of cytochromes (1585, 1316, and 750 cm^−1^) were the markers of the ER and mitochondrial features contributing to the clustering of the perilipidic area (Figure 2 and Figure S2, Table 1) [36]. In addition, the IR spectrum of the perilipidic area showed a unique signature of protein confirmations compared to the other classes (Figure S2B in Supplementary Materials). It exhibited the increased contributions of intermolecular β-sheets (1620 cm^−1^) and folded strands (1680 cm^−1^) compared to α-helices that could be associated with the accumulation of lipid-binding proteins [37].

The presence of sugars (IR: 1150, 1114, and 1050 cm^−1^) and amino acids (RS: 1007 and 643 cm^−1^; IR: 1396 cm^−1^) was also observed and correlated with the rough ER (RER) (Figure S2A,B in Supplementary Materials).

In turn, the Raman and FTIR spectra of the cytoplasm showed the unique features of proteins (RS: 2965–2850 and 1257 cm^−1^; IR: 2965–2850 and 1650 cm^−1^) with a dominance of α-helical secondary structure (Figure S2A,B in Supplementary Materials). Compared to other types of eukaryotic cells, the large pool of mitochondria in adipocytes supports their high metabolic functioning, as indicated by the increased intensity of the Raman signal of cytochromes in the cytoplasm (Figure S2A in Supplementary Materials).

The nucleus was identified based on the increased intensities of phosphates (1340, 1240, and 790 cm^−1^) and purines (1585, 1375, and 1316 cm^−1^), which are the characteristic Raman bands of nucleic acids (cf. Figure S2A and Table 1). This profile is similar to that of other eukaryotic cells [30].

### 2.4. An Effect of the Fixation Agents on the Chemical Composition of the Adipocytes

**Figure 4:**
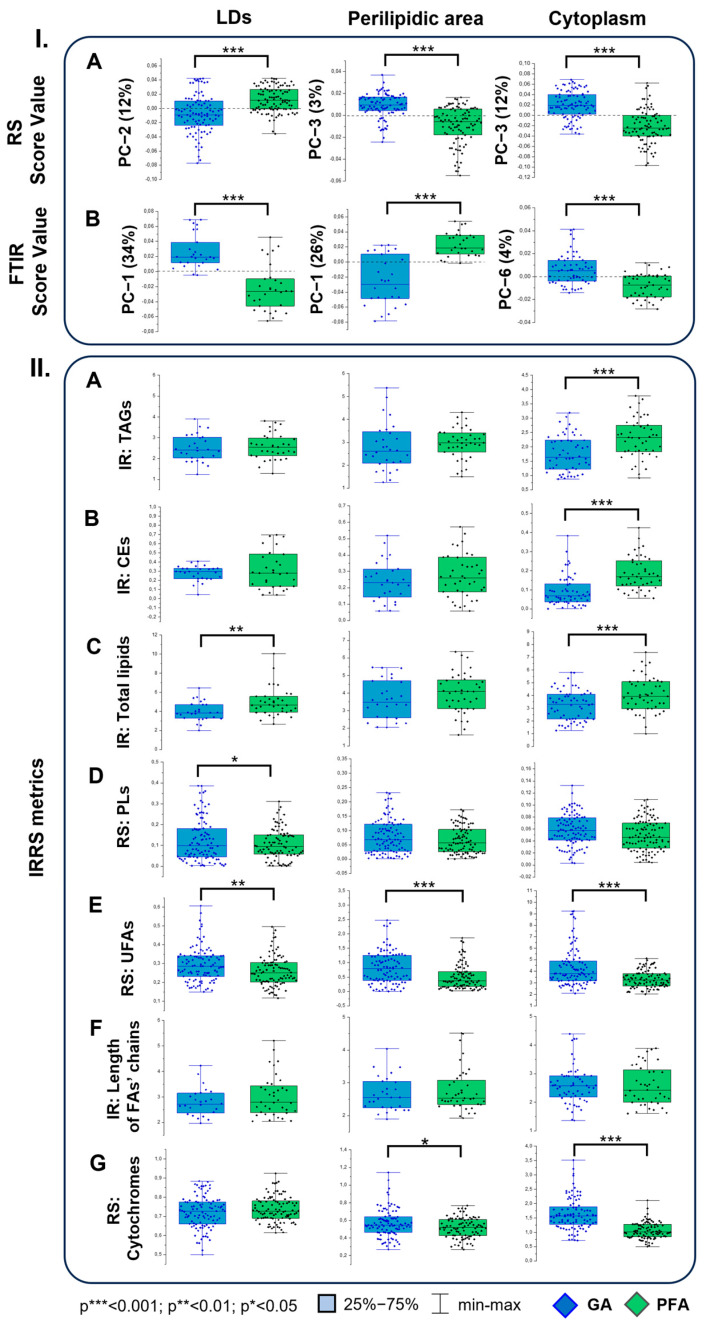
(**I**). The scores values from principal component analysis performed on (**A**) RS and (**B**) FTIR spectra of lipid droplets, perilipidic area, and cytoplasm of 3T3-L1-derived adipocytes preserved with glutaraldehyde (GA, in blue) and paraformaldehyde (PFA, in green). (**II**). Box diagrams representing semiquantitative analysis of lipid classes and cytochromes identified in the Raman (N = 100/fixative agent) and FTIR spectra (N = 30/fixative agent). (**A**) triacylglycerols—TAGs (IR: 1743/1465 cm^−1^), (**B**) cholesterol esters—CEs (IR: 1178/1465 cm^−1^), (**C**) the total content of lipids (IR: 2852 + 2954/1465 cm^−1^), (**D**) phospholipids—PLs (RS: 1129/1451 cm^−1^), (**E**) unsaturated fatty acids—UFAs (RS: 1660/1451 cm^−1^), (**F**) the length of acyl chain of FAs (IR: 2852/2954 cm^−1^), and (**G**) cytochromes (RS: 1585 + 1316 + 750/1451 cm^−1^). Each point refers to a single spectrum.

To portray the application of IRRS-based omics in adipocyte-related studies, here, we aimed to evaluate the compositional changes within fat cells (LDs, perilipidic area, and cytoplasm) resulting from different fixatives. As mentioned in the Introduction, paraformaldehyde and glutaraldehyde are the most often used agents. Using principal component analysis (PCA), which is very sensitive to spectra variation, and quantified intensities of the selected spectral markers (Figure 4), we estimated the effect of GA and PFA on the chemical composition of adipocytes. The graphs containing PCA loading vectors and the average RS and FTIR spectra from cellular compartments are depicted in Figures S3–S5 in the Supplementary Materials, respectively.

The RS and FTIR spectra of the LDs were separated along PC-2 and PC-1, respectively (Figure 4I(A,B)). The statistical significance of the semiquantitative analysis indicated the alternations of the content of some lipid classes within the lipid droplets, except the critical components of LDs—TAGs and CEs (Figure 4II(A,B), Table 1). It was apparent that the fixation of the cells with glutaraldehyde decreased the total lipids content estimated from absorbances of the high-wavenumber IR bands (Figure 4II(C)). Interestingly, the segregation of the RS and FTIR datasets of cytoplasm (PC-3 and PC-6, respectively, Figure 4I(A,B)) was also complemented with the decreased total content of lipids in the GA-fixed cells but with a simultaneous and significant decrease in TAGs and CEs (Figure 4II(A–C)). Likely, the membrane of the lipid droplet protects its interior from “leaking”; whereas, free sparse lipid classes in the cytoplasm are degraded. The perilipidic area did not reveal any differences in the content of lipids (Figure 4II(A–D)).

Other IR/RS metrics showed a PFA negative effect on the LD composition, as it induced the decomposition of PLs and UFAs (Figure 4II(D,E)). Both are well-known spectral markers of lipid peroxidation [38,39]. In the perilipidic area and cytoplasm, the loss of the unsaturation degree in FAs was even more significant (Figure 4II(E)), which was relevantly exhibited with the PCA score values (Figure 4I(A,B)). On the contrary, the level of phospholipids, organized in the form of intracellular bilayer membranes, did not show a detectable impact of GA and PFA (Figure 4II(D)). We assume that the monolayer membrane of LDs is considerably more liable than its bilayer equivalent for the detrimental effects of fixatives. Noteworthily, the length of the FA chains was not negatively affected by any of the fixatives in each compartment (Figure 4II(F)).

Lastly, a level of cytochromes correlated with mitochondria was estimated (Figure 4II(G)). The RS metrics of these hemoproteins showed that the mitochondria were substantially better preserved in the cytoplasm and perilipidic area of the GA-fixed cells. In the direct surroundings of LDs, no differences in the content of cytochromes were observed.

## 3. Discussion

The 3T3-L1 cell line is an in vitro model for studying the intracellular pathways involved during adipogenesis, since they are easily transformed in vitro from preadipocytes into mature adipocytes. This model is beneficial for investigating metabolic disorders, like obesity, insulin resistance, and type 2 diabetes [40,41]. During the differentiation process, preadipocytes lose their fibroblast-like shape and become mature adipocytes with a rounded morphology. The accumulation of a large number of lipid droplets is an indicator of differentiation.

For the adipocyte visualization, the Oil Red O staining remains a gold standard method, as it binds hydrophobic and neutral lipids, such as triglycerides, diacylglycerols, and cholesterol esters present in the interior of the lipid body. Contrarily, ORO does not identify membrane polar lipids, such as phospholipids, sphingolipids, and ceramides [42]. Moreover, as the ORO is dissolved in 60% isopropanol, it is very likely a primary reason for the fusion of small LDs [43]. As we showed in Figure 1A,C, the stained area strictly depends on the local morphology, varying in the median from 6.7 to 17.2%.

These outcomes highlight that the percentage of the ORO-stained area cannot be an unequivocal indicator of the advancement of adipogenic differentiation but rather the supporting information regarding the current condition of the cells and the presence of lipidic occlusions.

Another approach for depicting the adipocyte state is the IF labelling of FABP4. This cytoplasmic protein, expressed primarily in fat cells, acts as a transporter of intracellular lipids. As it binds fatty acids to biological targets, joins them to appropriate signaling pathways, and regulates glucose/lipid metabolism, the FABP4 is peculiar for LD formation in adipocytes due to induced differentiation [44]. FABP4 directly regulates the size of lipid droplets, and its higher concentration is correlated with the completion of the adipogenesis process, which may differ between the cells. The statistical analysis of the collected IF images revealed that, on average, 2.1% of their area is covered by the FABP4 signal (Figure 1F). Since the protein represents approximately 1% of all soluble proteins in adipocytes, its coverage is much lower than that of the determined area of the ORO staining [44]. Therefore, it cannot be unequivocally colocalized with the formed lipid bodies. Nevertheless, it is a crucial marker for detecting the ongoing maturation of preadipocytes (Figure 1A).

Images gathered by Raman microscopy with a spatial resolution of 0.3 µm (for the used here optics and laser excitation) were comparable with fluorescence microscopy. They probed the mature adipocytes’ intracellular compartments similarly, simultaneously providing chemical identification and morphological details (Figure 1D–F and Figure 2A–D). On the other hand, the separate identification of neutral lipids, like TAGs and CEs, impossible with the ORO staining, was unambiguously obtained by rapid and label-free IR spectroscopic imaging (Figure 1A and Figure 2A,E,F). Importantly, Raman and IR imaging also do not require labels that are dissolved in alcohol solvents, which causes the deformation of LDs and their fusion. In addition, the techniques were uniquely effective and fast to perform with a priori known spectral markers of the mature adipocytes, e.g., RS: 750 cm^−1^ band of cytochromes or IR: 1743 cm^−1^ band of TAGs, as the distribution maps can be constructed directly after the examination. Although high-resolution Raman imaging requires ca. 40 min to collect the image of a single mature adipocyte, data collection can be shortened up to 3 min through an increase in the step size (from 0.16 µm to 0.5 µm) and the reduction in the integration time (from 0.1 s to 0.01 s). Modifying these parameters alters the final localization of the cellular compartments. However, the accuracy of cluster assignment remains satisfactory, considering 10 times more rapid data acquisition (Figure S6 in Supplementary Materials). IR imaging, in turn, as a complementary technique in a chemical sense, required ca. 5 min to collect tens of the cells from a 700 µm × 700 µm area. Although we did not observe any morphological alternations in this case, one has to be aware that the air-drying process may disrupt the architecture of cellular compartments in other cell types. Nevertheless, the complement information carried by the RS and IR modalities can be acquired in minutes on both the subcellular and global levels.

Besides the morphological details provided by vibrational techniques, we also presented the biochemical information of the cellular structures of 3T3-L1-derived adipocytes. As a result of image segmentation, four classes of different profiles were derived and assigned to lipid droplets, the perilipidic area, cytoplasm, and the nucleus, respectively (Figure 2H).

The vibrational spectra of LDs indicated the presence of PLs, CEs, and TAGs rich in saturated and monounsaturated fatty acids (RS), with an averaged length corresponding to trilaurin (36:0, IR) (Figure 3). One should carefully assign the value of the CH_2_:CH_3_ ratio to a particular lipid, since it illustrates all lipid species present in LDs. Still, it is a good estimator of the acyl chain length. The RS-based observation was congruent with a lipidomics-based study on the differentiated 3T3-L1 cells, reporting that the LD membrane of these cells primarily consists of C16:0, C16:1, C18:1, and C18:1 FAs [45]. Lipidomic phenotyping of human adipocytes reported that the LD composition became more homogeneous through adipogenic differentiation, with highly concentrated SFAs and MUFAs, with the four FAs mentioned above as the most abundant [46].

As mentioned above, another illustrated cellular compartment was the perilipidic area. This thin layer coating the lipid droplets corresponded morphologically to LD membranes and lipid-droplet-binding proteins, like perilipins and FAPB4. Among the former, perilipin 1 is restricted to LDs and acts as their scaffold, mediating protein–protein interactions [47]. In addition, an extensive study on the role of LD-binding proteins has shown that a portion of perilipin 1 overlaps the ER in various adipocytes, including the 3T3-L1 cell line, and it is an indicator of the bidirectional traffic between the ER and LDs [48]. In turn, FABP4 secretion and FA transport are connected with mitochondrial activity pronounced in the differentiation process to adipocytes [49]. For this observation, multiplex immunochemical staining and Western blotting are required. Here, the RS and IR spectra of the PA indicated its lipidic profile, but rich in phospholipids, cytochromes, sugars, and amino acids comparable to the LD composition (Figure 3 and Figure S2, Table 1). Notably, the abundance of these biomolecules was correlated with mitochondria and ER, particularly RER [36]. The RER has many roles in protein synthesis, including post-translational modifications, folding, and sorting. Membrane-bound ribosomes in the RER translate the mature mRNA transcript into amino acids that are attached to then become polypeptides [50]. In addition, the accumulation of lipid-binding proteins resulted in a unique signature of protein confirmations compared to the other classes (IR, Figure S2B in Supplementary Materials) [37]. As such, with the combination of IR and RS approaches (IRRS-based omics), it is possible to track, in a label free way, the interactions between LDs and ER, the metabolic activity, and molecular modification within the interior of adipocyte cells, enabling the further development of research concerning fat cell pathologies.

As the proof-of-concept for applying IRRS-based omics in adipocyte-related studies, we investigated the compositional changes of fat cell structures (LDs, perilipidic area, and cytoplasm) in response to different fixatives. As mentioned in the Introduction, the reports published so far indicated that selecting the fixative agent for cell preservation is crucial for the LD’s size and the visualization of proteins by histological and immunochemical staining. In this study, the mature adipocytes were fixed with those frequently used in microscopy: 4% paraformaldehyde or 2.5% glutaraldehyde (Figure 4) [9,13,43]. Using principal component analysis and quantified intensities of the selected spectral markers, it was possible to estimate the effect of GA and PFA on the chemical composition of adipocytes. Noteworthily, PCA, as a multivariate method, is already well-known within the metabolic/lipidomic field through its facilitation of data representation [51].

The statistically significant segregations of datasets were followed with the semiquantitative analysis, indicating the alternations of the content of some lipid classes within lipid droplets, except the critical components of LDs—TAGs and CEs (Figure 4II(A,B), Table 1). It was apparent that the fixation of the cells with glutaraldehyde decreased the total lipid content. In our previous spectroscopic study on the fixation effect of the cells, we observed a similar impact of 1 and 2% GA on erythrocytes and their membranes [38]. Since the GA treatment did not affect the TAG and CE levels in LDs, we imply that the total lipid decrease was due to increased membrane permeability and the washing out of lipids induced by this agent. Since we used the transmission FTIR mode, the spectrum detects this change. Other IR/RS metrics showed a PFA negative effect on the LD composition (Figure 4II(D,E)). However, this agent is preferable for preserving the cytoskeleton and immunolabeling LD-related proteins [9]. PFA induced the decomposition of PLs and UFAs. Both are well-known spectral markers of lipid peroxidation [38,39]. Thin-layer chromatography of lipid extracts from adipocytes fixed with PFA confirmed our semiquantitative analysis, i.e., the content of TAGs and CEs remains constant. In contrast, the level of phospholipids decreases by ca. 10% [9]. On the contrary, the level of phospholipids, organized in the form of intracellular bilayer membranes, did not show a detectable impact on GA and PFA (Figure 4II(D)). We assume that the monolayer membrane of LDs is considerably more liable than its bilayer equivalent for the detrimental effects of fixatives. Lastly, the contribution of cytochromes correlated with mitochondria was estimated (Figure 4II(G)), and these organelles were substantially better preserved in the cytoplasm and perilipidic area of the GA-fixed cells. This observation is consistent with an IF-based study on fixative methods suitable for preserving mitochondria, which found that glutaraldehyde maintains the morphology of this organelle [52].

To sum up the obtained results, a few conclusions should be highlighted. Firstly, high-resolution Raman imaging of the single cells recognizes substructures of the fat cells, like the nucleus and its perinuclear area, representing the endoplasmic reticulum, cytoplasm, and finally—the main player in the metabolism of fat cells—lipids droplets surrounded by the directly associated proteins. Their presence was confirmed by the corresponding staining, meaning that Raman images can be used for morphology analysis, not only for lipid droplets as seen so far. To the best of our knowledge, no report has shown such a clear-cut assignment of these structures to their unique Raman signature or given an interpretation for further investigations, e.g., phospholipids in the LD membrane and cytochromes in the cytoplasm. The nucleus was segregated similarly to other cells, and its dysfunction, if any, can observed by content changes in phosphate moieties and purines. Next, IR imaging, despite the loss of the single-cell detection, is capable of recognizing the key elements of the fat cells, like LDs, their associated proteins, perilipidic area, and cytoplasm, by rapidly scanning a large area. The proof of the identification of these features was possible by Raman imaging, firstly, and this IR pattern should be applicable in further investigations of a cell with lipid bodies. The unprecedent capability of FTIR spectroscopy is its identification of the secondary conformations of proteins and sugars that were uniquely detected in an area around LDs and were attributed to abundant proteins in those cellular structures. Their observation will deliver the information about the disruption of the LD physiology and formation. Our finding has not been reported so far. The last point is our proof-of-principle spectral lipidomics. The plethora of lipids detected by both spectroscopies delivered the possibility of the quantification of the total content of lipids, FAs, UFAs, TAGs, CEs, PLs, and the length FA acyl chains. Most reports listed in the Introduction were focused on the saturation degree and the TAG level. We showed here the possibility of the determination of the calibration curves for the unsaturation degree (RS) and lengths of the fatty acids (IR). While the former has been proposed in several reports, the latter has not been considered until now. These examples showed the opportunity of the quantification of all the identified lipid classes, but one should be aware that the result is an average value for a given lipidic class. Very likely, the combination of regression analysis of the spectral and MS data for the same sample will reliably evaluate in situ IR/RS predictors of the LD content. This results from the drawback of the used spectroscopy that does not precisely recognize particular lipids if several specific compounds contribute to the composition of LDs. However, this approach appears as much easier to obtain when comparing to the HPLC/MS lipidomics that require a large amount of the sample, and is unable to attribute the lipid origin to the cellular compartments. Finally, we claim that this methodology is appropriate for probing fat cells regardless of their origin, since their spectral features are expected to be preserved in each cell type exhibiting these structures.

## 4. Materials and Methods

### 4.1. Chemicals

DMEM 4.5 g/L D-glucose medium and calf serum (CS) were purchased from ATCC: The Global Bioresource Center (Manassas, VA, USA). Fetal bovine serum—FBS, Blocker BSA in TBS, Penicillin/Streptomycin Solution, Bovine Serum Albumine—BSA and 4% paraformaldehyde in PBS, Secondary Donkey anti-Goat antibody, and AF555 conjugated (#A-21432) were purchased from Thermo Fisher Scientific (Waltham, MA, USA), Dulbecco’s Phosphate Buffered Saline—PBS (Corning, NY, USA), 3-Isobutyl-1-methylxanthine—IBMX, dexamethasone, insulin, Oil Red O solution in 60% isopropanol, Poli-L-lysine, bisBenzamide H 33342 trihydrochloride, and Phalloidin Atto 488 were purchased from Sigma Aldrich (Stenheim, Germany). Anti-mFABP4 primary antibody (R&D Systems, Minneapolis, MN, USA), Vector TrueVIEW Autofluorescence Quenching Kit, VECTASHIELD Vibrance Antifade Mounting Medium with DAPI, and glutaraldehyde—GA 25% solution in water were purchased from Biokom (Janki, Poland).

### 4.2. Cell Line

This study used a 3T3-L1 preadipocyte cell line derived from mouse embryonic cells (ATCC, Manassas, VA, USA). Cells were cultured in DMEM 4.5 g/L D-Glucose (ATCC, Manassas, VA, USA) supplemented with 10% CS (ATCC, Manassas, VA, USA) and Penicillin/Streptomycin Solution (Gibco, Waltham, MA, USA). The cells were cultured at 37 °C in a humidified atmosphere with 5% CO_2_ and were passaged every 3–4 days. Cells between the 3rd and 7th passages were used for the experiment.

### 4.3. 3T3-L1 Adipogenesis

The 3T3-L1 cells in the logarithmic growth phase were seeded into 24-well plates (Falcon, Corning, NY, USA) at a concentration of 3 × 10^4^ cells/mL/well in DMEM 4.5 g/L D-Glucose medium supplemented with 10% CS. The wells contained sterile 12 mm CaF_2_ slides (Crystran, Poole, UK) required for spectroscopic imaging. After reaching confluence (48–72 h), the cells were cultured in a growth medium (DMEM + 10% CS) for 48 h. A differentiation medium containing DMEM 4.5 g/L D-Glucose, 10% FBS (Gibco, Waltham, MA, USA), 0.5 mM IBMX (Sigma Aldrich, Stenheim, Germany), 1 µM dexamethasone (Sigma Aldrich, Stenheim, Germany), and 15 µg/mL insulin (Sigma Aldrich, Stenheim, Germany) were added to the cells in the next step [53]. Next, an insulin-containing medium (DMEM 4.5 g/L D-Glucose, 10% FBS, 15 µg/mL insulin) was added for 48 h. Subsequently, a growth medium was added, and the cells were cultured for 72 h.

### 4.4. Cells Fixation

For spectroscopic analysis and histological (Oil Red O) and immunofluorescence staining, cells were fixed with 4% PFA (Thermo Fisher Scientific, Waltham, MA, USA) in PBS (30 min, RT) following the differentiation process (7 days). An additional batch of differentiated cells fixed with 2.5% GA (SERVA, Heidelberg, Germany) in PBS (2 h, 4 °C) were prepared for spectroscopic comparison of PFA- and GA-based fixations. The fixed cells were washed three times with PBS and left at 4 °C for further measurements.

### 4.5. Histological and Immunofluorescence Staining and Actin Labeling

Histological Oil Red O staining was performed using a 0.3% solution of Oil Red O in 60% isopropanol. Cells were preincubated in 60% isopropanol (30 min, RT) following Oil Red O incubation (60 min, RT) and washed four times with sterile deionized water. The images were recorded by a Zeiss Axio Observer microscope with a Zeiss AxioCam 506 color camera. ZEN Blue Edition v 3.4 software (Zeiss, Oberkochen, Germany) was used for the analysis; whereas, the total stained area size of LDs was determined using ImageJ software (v. 1.54d, NIH, University of Wisconsin, Madison, WI, USA).

For the immunohistochemical localization of FABP4 protein, 3T3-L1 cells were seeded on 4-well Lab Tek II (Thermo Fisher Scientific, Waltham, MA, USA) coated with 0.1 mg/mL Poly-L-lysine (Sigma Aldrich, Stenheim, Germany), dissolved in PBS at 3 × 10^4^ cells/mL/well in growth medium. Cells were differentiated as described above. Fixed 3T3-L1 cells were washed three times with 0.1% BSA solution in PBS and then permeabilized and blocked with Blocker BSA in TBS for 1 h in RT. Anti-mFABP4 primary antibody (to visualize this LDs-associated protein localization), diluted in 0.1% BSA (1:50) in PBS, was added for overnight incubation at 2–8 °C. After incubation, the cells were washed three times with PBS. Next, donkey anti-Goat IgG secondary antibody conjugated with AlexaFluor 555 was added (60 min, RT, in the dark). In addition, for actin localization, cells were washed three times with PBS and incubated with Phalloidin Atto 488 (decorating actin cytoskeleton) for 1 h at RT in darkness, diluted 1:50 in 0.1% BSA in PBS. bisBenzamide H 33342 trihydrochloride diluted 1:1000 in PBS was used to stain cell nuclei (15 min, RT, in the dark). A Vector TrueVIEW Autofluorescence Quenching Kit was used to quench the nonspecific fluorescence (1 h, RT, in the dark). VECTASHIELD Vibrance Antifade Mounting Medium with DAPI was used to close chamber slides. The Zeiss Axio Observer microscope equipped with an AxioCam 530 monochromatic camera and ZEN Blue Edition v 3.4 (Zeiss, Oberkochen, Germany) were used for imaging. A further analysis (nuclei and FABP4 areas) was performed using the ImageJ software (v. 1.54d).

### 4.6. Raman and FTIR Microscopy

Raman imaging was carried out using a WITec confocal Raman imaging system (a WITec alpha 300 Raman microscope, WITec GMBH, Ulm, Germany). Raman spectra were acquired with an excitation laser at 532 nm and an integration time of 0.01 s. The microscope was equipped with a CCD detector cooled to −80 °C. Cells adhered to CaF_2_ windows and immersed in PBS solution were illuminated through a 60× water immersive Zeiss objective (NA: 1.0). Raman images (ca. 100 cells per the experimental group) were recorded with a step size of 0.5 μm. Single cells were additionally imaged using high-resolution scanning (HR) with a step size of 0.16 μm and an integration time of 0.1 s. In this case, a maximum lateral resolution of 0.3 μm was achieved for the used optical setup. All spectra were collected with a spectral resolution of 3 cm^−1^.

IR images were acquired using an Agilent 670-IR FTIR spectrometer connected with a 620-IR microscope (Agilent, Santa Clara, CA, USA) and coupled with a focal plane array (FPA) detector. The detector consisted of a matrix of 16,384 pixels, arranged in 128 × 128 grid format. IR images were acquired in transmission mode using a 15× objective and condenser optics with NA of 0.62 and a projected FPA pixel size of 5.5 μm × 5.5 μm (Standard Definition mode, SD, maximum lateral resolution of 6–10 μm in the 1000–1800 cm^−1^ region). FTIR spectra of cells were recorded by co-adding 64 scans (256 scans for background) in the range of 900–3700 cm^−1^ with a spectral resolution of 4 cm^−1^. For each experimental group, 30 images (each 700 μm × 700 μm) were acquired to cover the representative region of cells. Reference lipids were measured with the same spectral parameters.

### 4.7. Raman and FTIR Data Analysis

The initial preprocessing of Raman images was carried out using Project Five 5.3 software. Firstly, cosmic rays were removed with a filter size of 3 and a dynamic factor of 8. Then, the spectral baseline was adjusted with a polynomial of the 3rd order. To visualize biocomponents detected by Raman scattering, chemical maps of long-chain fatty acids (2830–2900 cm^−1^), nucleic acids (780–800 cm^−1^), and cytochromes (740–760 cm^−1^) were constructed. Finally, *k*-means cluster analysis (KMCA) analysis was performed to discriminate four representative classes of cellular compartments, i.e., lipid droplets, the perilipidic area, the nucleus, and cytoplasm.

The extracted mean KMCA spectra were preprocessed using Opus 7.0 software. To moderate the impact of autofluorescence, a baseline correction was applied using a concave rubber band correction method (10 iterations). Then, the corrected spectra were smoothed according to a Savitzky–Golay protocol (11 points), cut to 500–3100 cm^−1^, and vector normalized.

For the preprocessing of IR images, CytoSpec (v. 2.00.04) and MATLAB (v. R2015a) software were employed [54]. Firstly, the distribution maps of proteins (1620–1680 cm^−1^), triacylglycerols (1720–1770 cm^−1^), and cholesteryl esters (1160–1190 cm^−1^) were constructed to visualize cytoplasm and lipid accumulation. Before clustering, the quality test was employed to eliminate the signal with an absorbance lower than 0.2. This operation was performed in the region between 1620 and 1680 cm^−1^. To remove spectral noise, PCA-based noise reduction with 13 PCs was executed. Next, the spectra were smoothed with 13 smoothing points according to the Savitzky–Golay protocol and vector normalized to exclude the effect of the sample thickness. Unsupervised hierarchical cluster analysis (UHCA) was executed in the spectral regions of 900–1800 and 2800–3050 cm^−1^. Spectral distances were computed as D-values, and individual clusters were extracted according to Ward’s algorithm. The number of classes was adjusted to achieve the maximum differentiation of FTIR spectra and to discriminate the lipid droplets, the perilipidic area, and cytoplasm. The mean FTIR spectra and cluster maps were extracted from the calculated UHCA classes.

Finally, resonant Mie scattering (RMieS) correction using seven principal components was performed on all extracted FTIR spectra [55]. The Opus 7.0 software calculated the second derivative of the FTIR spectra with 13 smoothing points according to the Savitzky–Golay protocol.

Afterward, the RS and FTIR spectra of the cellular compartments were averaged and compared.

### 4.8. Semiquantitative and Principal Component Analysis

The integral intensities of Raman and IR bands in the preprocessed spectra were calculated as an area under the band contour (OPUS 7.0). Then, their box charts were constructed. An analysis of variance was performed using an ANOVA statistical model, while statistical significance (*p*-values) was determined by a Tukey’s test on the levels of *p* < 0.05, *p* < 0.01, and *p* < 0.001.

Principal component analysis (PCA) was performed on the spectra obtained in the cluster analysis using Unscrambler X 10.3 software (CAMO Software AS, Oslo, Norway). Spectral preprocessing included smoothing, offset baseline correction, vector normalization, and mean-centering (weight of 1.0). PCA was performed in the spectral bioregion of 1000–3050 cm^−1^ (FTIR) and 550–3050 cm^−1^ (RS), excluding the silent region (1800–2800 cm^−1^), and with the use of a NIPALS algorithm (random cross-validation with 20 segments) for seven principal components. Score and loading plots were graphed using Origin 2021b software.

## 5. Conclusions

Here, we reported a label-free analytical method based on Raman and FTIR spectroscopy imaging as an excellent alternative to standard histological and immunohistochemical staining applied in studies on cells containing lipid droplets. It is suitable for examining the morphology of LDs, since Raman imaging recognizes intracellular structures with a diameter greater than 2 μm in single cells. In contrast, FTIR imaging shows the heterogeneous distribution of accumulated lipids in the large area of the cellular films. The primary aim of probing the cells with these techniques is to determine lipid classes present in LDs without extensive sample preparation and in the naïve state. We proved that both spectra provide distinct chemical information about lipids, e.g., the esterification of fatty acids and cholesterol (IR), the unsaturation degree of the acyl chain, and phospholipids (RS), and can serve as the preliminary lipidomic assay. Moreover, we showed that the cellular environment around the lipid bodies can be detected simultaneously, and the biomolecules related to the cell function were indicated. Finally, we provided a list of the specific spectral markers of each compartment discriminated from the chemical images of the cell body. In that way, IR and Raman microscopies can complement or even substitute optical and histological examination of the fat cells, revealing a set of morphological and chemical properties without tags and extraction and following them in LD-linked dysfunctions.

## Figures and Tables

**Table 1:** Raman and FTIR bands specific for the cellular compartments of 3T3-L1-derived adipocytes. The vibrational modes of bands are listed in Tables S1 and S2 in Supplementary Materials.

	Raman Spectra		FTIR Spectra	
	Band Position [cm^−1^]	Biomolecules	Band Position [cm^−1^]	Biomolecules
LDs	1070, 1451, 2855 896 1270, 1306, 1660, 3010 1099, 1129 605 1743	FAs SFAs UFAs PLs CEs TAGs	1465, 2852 - 3010 - 1178 1743	FAs - UFAs - CEs TAGs
Features of lipids	1660/1451 - -	Unsaturation degree - -	- 2852 + 2954/1465 2852/2954	- Total lipids Length of FA chains
Perilipidic area	1099, 1129 750, 1316, 1585 - - - 643, 1007	PLs Cytochromes - - - AAs	1240, 1340 - 1620 1680 1150, 1114, 1050 -	PLs - Intermolecular β-sheets Folded strands in proteins Sugars -
Cytoplasm	1257 - -	Proteins - -	1650 1396 1080	α-helices in proteins AAs Nucleic acids
Nucleus	790, 1240, 1340 1316, 1375, 1585	Phosphates Purines (A, G)	- -	- -

FAs—fatty acids; SFAs—saturated fatty acids, UFAs—unsaturated fatty acids; PLs—phospholipids; CEs—cholesteryl esters; TAGs—triacylglycerols; AAs—amino acids; A—adenine; G—guanine.

## Data Availability

Data will be made available on request.

## Supplementary Materials

The following supporting information can be downloaded at: https://www.mdpi.com/article/10.3390/ijms252212274/s1.
