# Supplementary material for: Chemical Landscape of Adipocytes Derived from 3T3-L1 Cells Investigated by Fourier Transform Infrared and Raman Spectroscopies
Source: Int J Mol Sci. 2024 Nov 15;25(22):12274. doi: 10.3390/ijms252212274 (PMC11595028; doi:10.3390/ijms252212274)
Supplement: Supplementary file 1 [file ijms-25-12274-s001.zip › ijms-3304426-supplementary.pdf]

Supplementary Materials

Chemical Landscape of Adipocytes Derived from 3T3-L1 Cells Investigated by Fourier Transform Infrared and Raman Spectroscopies

Karolina Augustyniak [1,2], Monika Lesniak [3], Maciej P. Golan [3,4], Hubert Latka [1], Katarzyna Wojtan [1], Robert Zdanowski [3], Jacek Z. Kubiak [3,5,*] and Kamilla Malek [1,*]

[1] Faculty of Chemistry, Jagiellonian University, Gronostajowa 2, 30-387 Krakow, Poland; karolina.augustyniak@doctoral.uj.edu.pl (K.A.); hubert.latka99@gmail.com (H.L.); k.wojtan@student.uj.edu.pl (K.W.)

[2] Doctoral School of Exact and Natural Sciences, Jagiellonian University, Prof. Stanislawa Lojasiewicza 11, 30-348 Krakow, Poland

[3] Laboratory of Molecular Oncology and Innovative Therapies, Military Institute of Medicine—National Research Institute, Szaserow 128, 04-141 Warszawa, Poland; mlesniak@wim.mil.pl (M.L.); mgolan@aps.edu.pl (M.P.G.); rzdanowski@wim.mil.pl (R.Z.)

[4] Institute of Psychology, The Maria Grzegorzewska University, Szczesliwicka 40, 02-353 Warsaw, Poland

[5] Dynamics and Mechanics of Epithelia Group, Institute of Genetics and Development of Rennes (IGDR), French National Centre for Scientific Research (CNRS), Faculty of Medicine, University of Rennes, UMR 6290, 35043 Rennes, France

* Correspondence: jacek.kubiak@univ-rennes.fr (J.Z.K.); kamilla.malek@uj.edu.pl (K.M.)

Table S1. RS band positions and their assignments to vibrational modes of biomolecules [1–8].

Band position [cm^{-1}]	*Assignment to biomolecules and vibrational modes*
**605**	Cholesterol, cholesterol esters; ν(steroid ring)
**643**	Tyr (proteins); δ(C-C)
**750**	Cyt. c and c_1; ν(porphyrin ring)
**790**	Nucleic acids; $\nu_s(PO_2)$
**896**	Proteins; $\delta(CH_2)$
**1007**	Phe (proteins); ring breathing
**1040**	Cross-linked Phe (proteins)
**1070**	Fatty acids; ν(C-C)
**1099**	Phosphate-containing molecules; $\nu_s(PO_2)$
**1129**	Phospholipids; ν(C-C)<br>*Cyt. c and c_1; ν(porphyrin ring)
**1240**	Phosphate-containing molecules; $\nu_{as}(PO_2)$
**1257**	Proteins (amide III); ν(C-N), δ(N-H), $\nu(CH_3\text{-}C)$
**1270**	Unsaturated lipids; δ(=CH)
**1306**	Unsaturated fatty acids; $\tau(CH_2/CH_3)$
**1316**	G (nucleic acids); ring breathing, δ(C-H)<br>*Cyt. c and c_1; δ(C-H)
**1340**	Nucleic acids, proteins; ring breathing; δ(C-H)<br>Lipids; $\tau/\delta(CH_2/CH_3)$
**1375**	A (nucleic acids); $\delta(CH_3)$
**1451**	Proteins, lipids; $\delta(CH_2, CH_3)$
**1585**	A, G (nucleic acids); ring breathing<br>*Reduced cyt. c, c_1 and b; ν(methine bridges – C_aC_m,C_aC_mH bonds)
**1660**	Unsaturated fatty acids; ν(C=C)
**1743**	Triacylglycerols; ν_{ester}(C=O)
**2855**	Long chain fatty acids; $\nu_s(CH_2)$
**3010**	Unsaturated fatty acids; ν(=C-H)

ν – stretching mode, as – asymmetric, s – symmetric; δ – in-plane deformations; ; τ – twisting; cyt – cytochromes; A – adenine; G – guanine; Tyr – tyrosine; Phe – phenylalanine. *Bands at 1130, 1311, and 1587 cm^{-1} are assigned to cytochromes only if the ~750 cm^{-1} band is present.

Table S2. FTIR band positions and their assignments to vibrational modes of biomolecules [9–14].

Band position [cm^{-1}]	*Assignment to biomolecules and vibrational modes*
**1050**	Poly/sugars; ν(CC-OC)
**1080**	Nucleic acids; $\nu_s(PO_2^-)$<br>Phospholipids; $\nu_s(PO_2^-)$
**1114**	Ribose (RNA); ν(C-O)<br>Poly/sugars; ν(CC-OC)
**1150**	Poly/sugars; ν(CC-OC)
**1178**	Cholesterol esters; ν_{as}(CO-O-C)
**1240**	Phospholipids; $\nu_{as}(PO_2^-)$
**1340**	Phospholipids, fatty acids, triacylglycerols; $\nu(CH_2)$
**1396**	Free amino acids; $\nu_s(COO^-)$
**1465**	Fatty acids; $\delta(CH_2, CH_3)$
**1620**	Intercellular aggregations of cellular proteins
**1650**	α-helices in proteins (amide I); ν(C=O) and δ(N-H)
**1680**	β-turns in proteins (amide I); ν(C=O) and δ(N-H)
**1743**	Triacylglycerols; ν_{ester}(C=O)
**2852**	Long chain FAs; $\nu_s(CH_2)$
**2954**	Lipids and proteins; $\nu_{as}(CH_3)$
**3010**	Unsaturated lipids; ν_s(=CH)

ν – stretching mode, as – asymmetric, s – symmetric; δ – in-plane deformations.

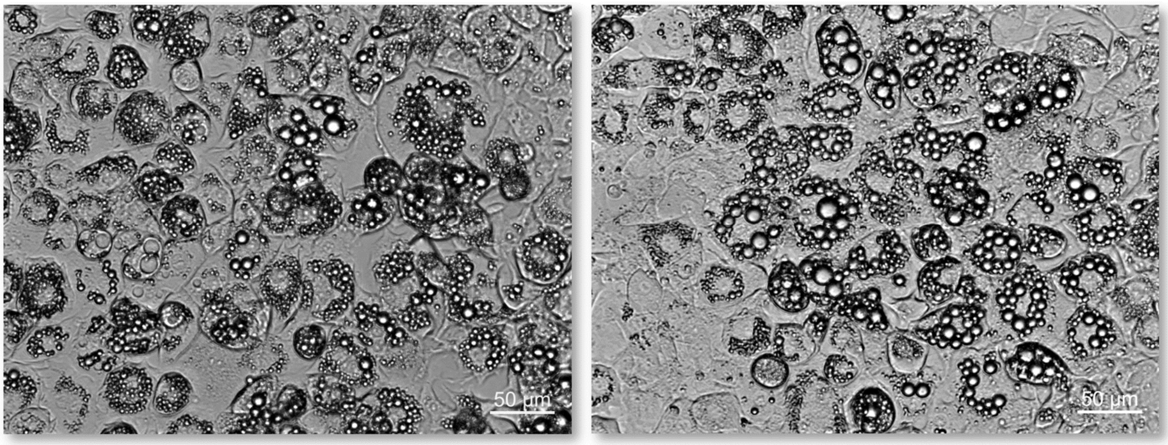

Figure S1. Exemplary bright-field optical images of 3T3-L1 derived adipocytes.

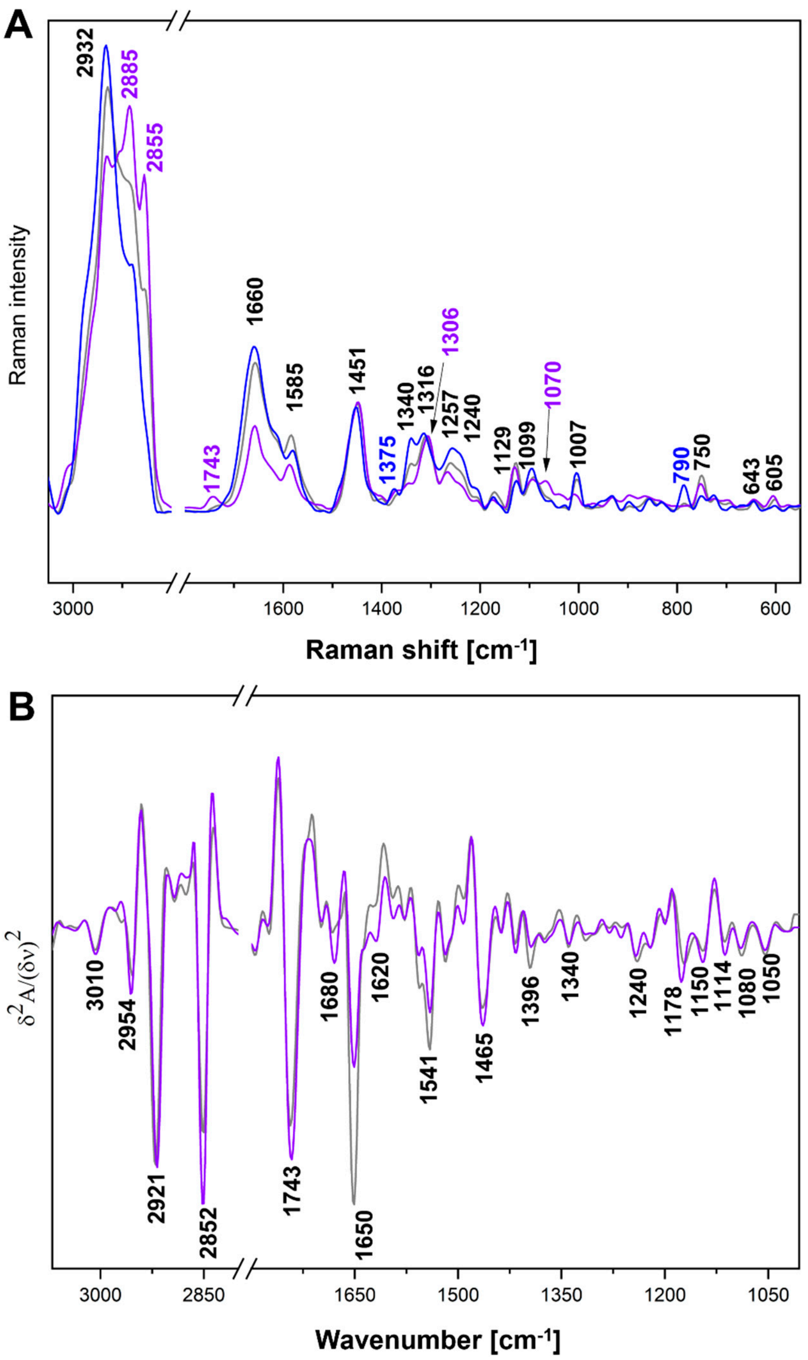

Figure S2. (A) The averaged Raman (N = 100) and **(B)** FTIR (N = 30) spectra of perilipidic area (purple), cytoplasm (gray), and nucleus (blue) from PFA-fixed adipocytes in the spectral regions of 3050-550 cm^{-1} and 3070-1005 cm^{-1}, respectively.

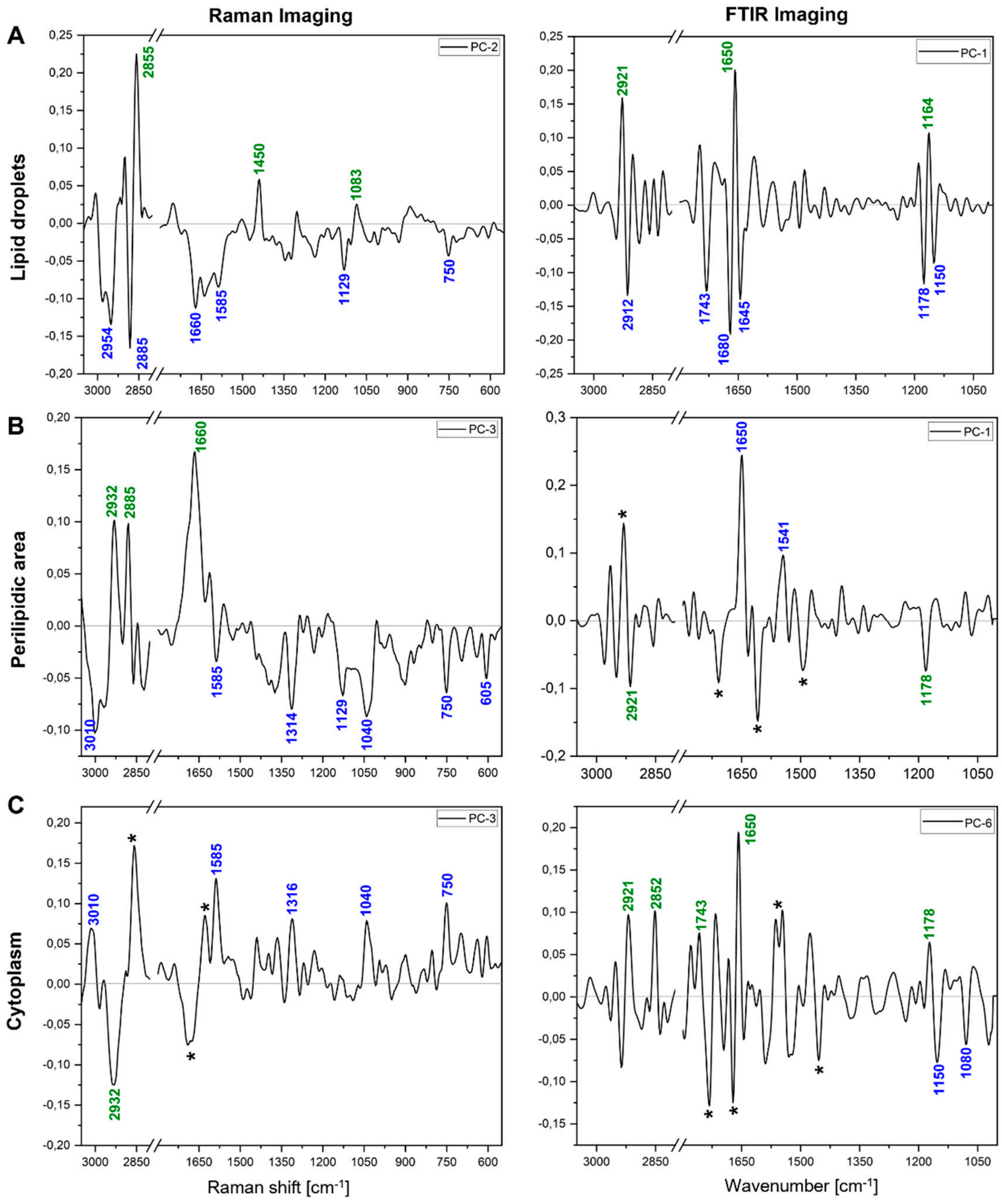

Figure S3. The PCA loading graphs of RS and FTIR datasets in the spectral regions of 3050-550 cm^{-1} and 3070-1005 cm^{-1}, respectively. **(A)** lipid droplets, **(B)** perilipidic area, and **(C)** cytoplasm from GA- (in blue) and PFA-fixed (in green) adipocytes. Artifacts marked with asterisks.

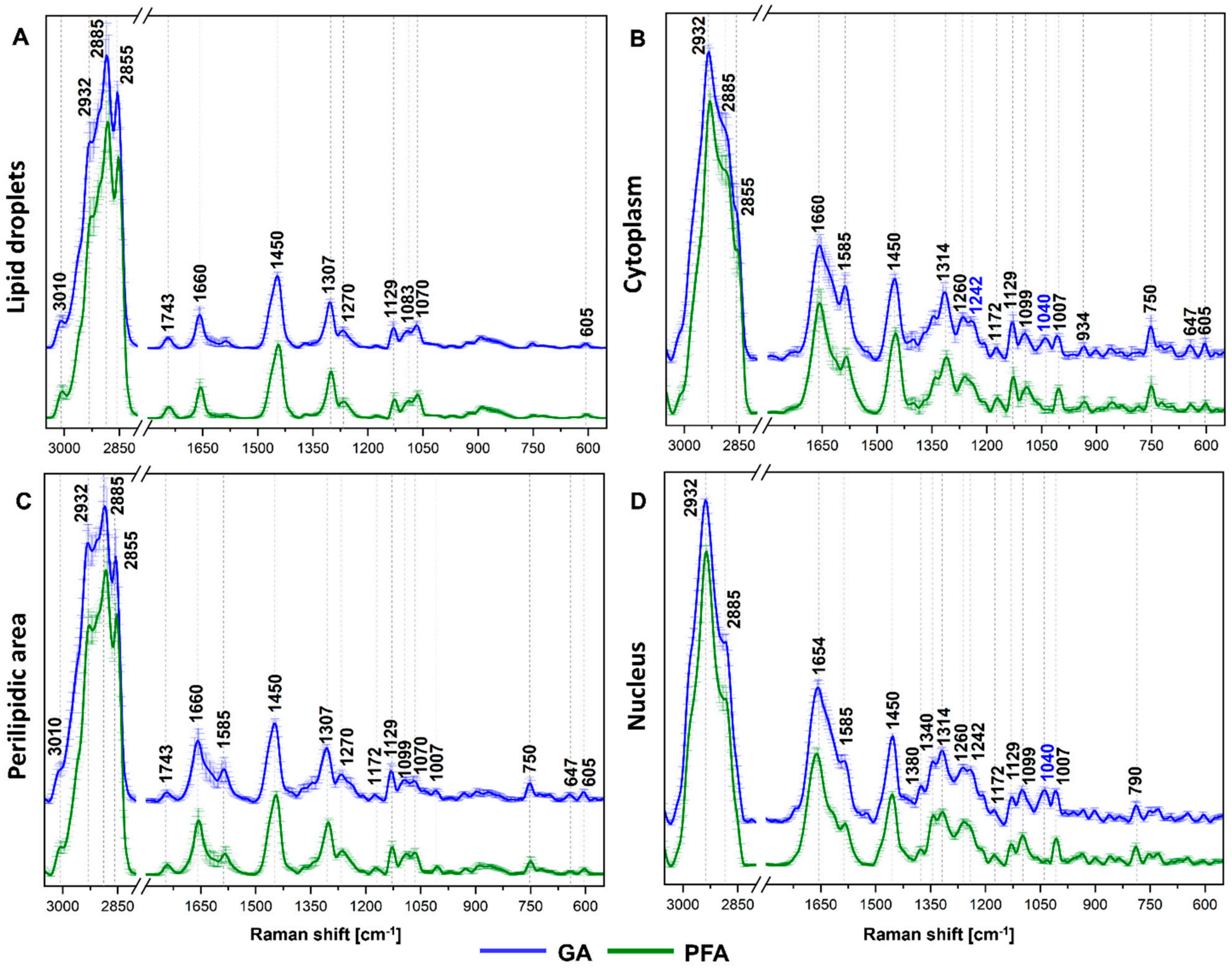

Figure S4. Comparison of averaged Raman spectra of **(A)** lipid droplets, **(B)** cytoplasm, **(C)** perilipidic area, and **(D)** nucleus, obtained from the differentiated 3T3-L1 cells fixed with glutaraldehyde (blue) and paraformaldehyde (green). Shade represents the ± standard deviation.

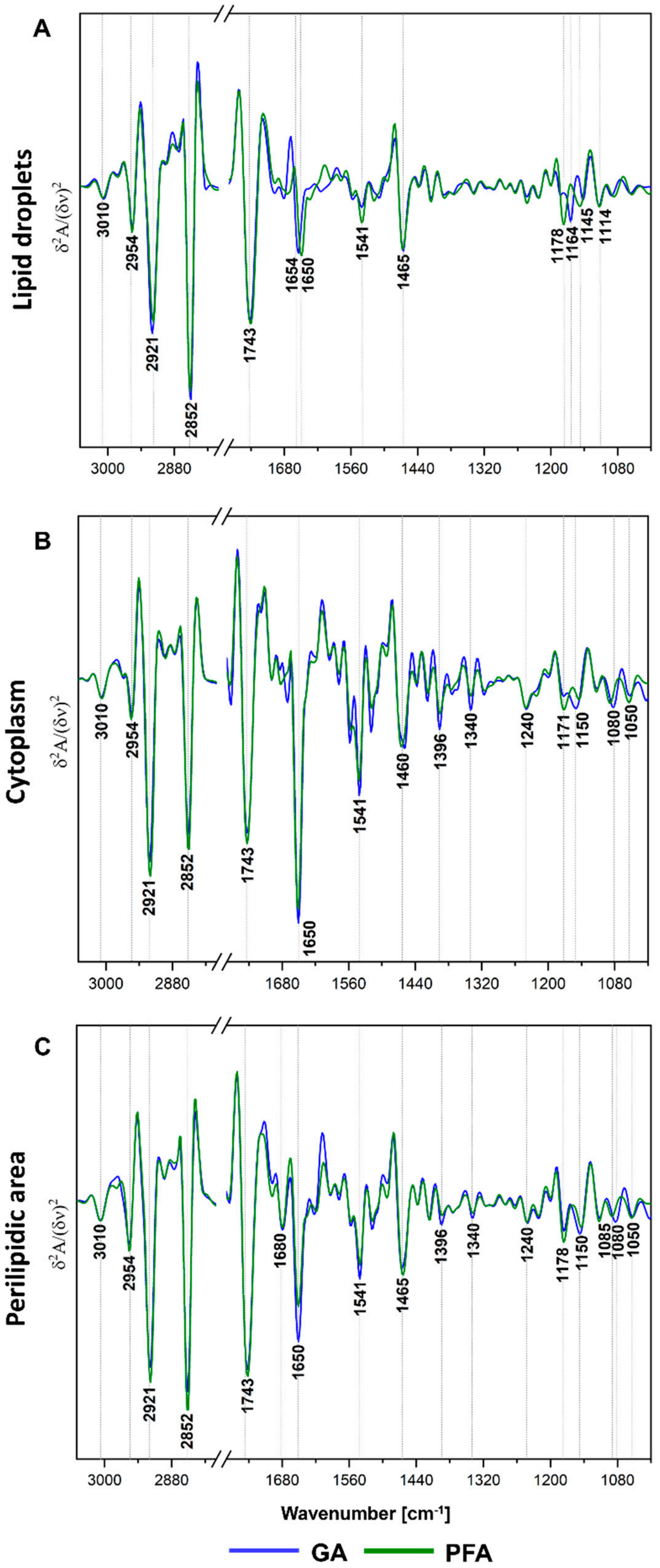

Figure S5. Comparison of averaged FTIR spectra of **(A)** lipid droplets, **(B)** cytoplasm, and **(C)** perilipidic area, obtained from the differentiated 3T3-L1 cells fixed with glutaraldehyde (blue) and paraformaldehyde (green).

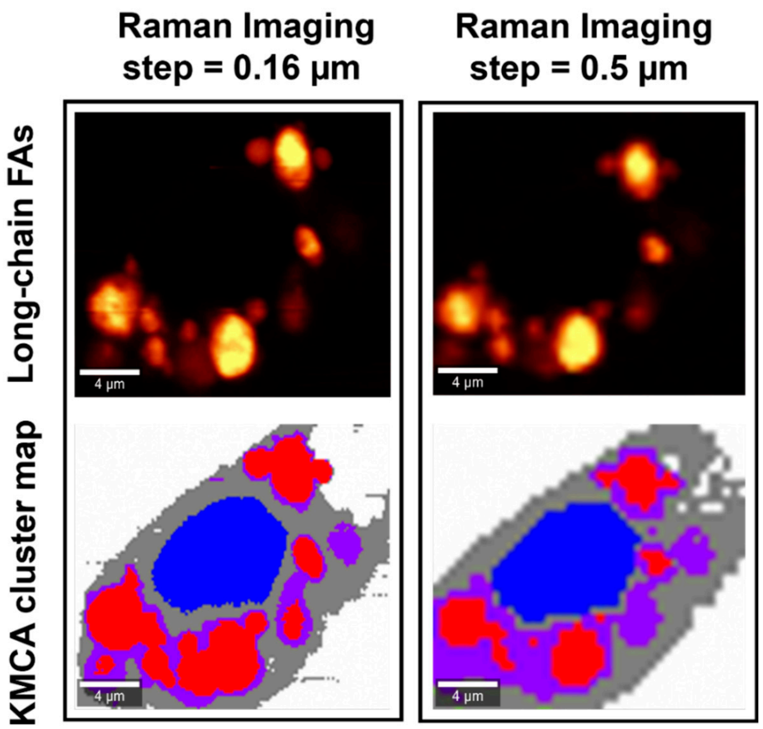

Figure S6. Distribution maps of long-chain fatty acids constructed from Raman images acquired with 0.16 µm and 0.5 µm step sizes, respectively. KMCA segregated the cell interior into the same compartments with acceptable accuracy.

1. Bik, E.; Dorosz, A.; Mateuszuk, L.; Baranska, M.; Majzner, K. Fixed versus Live Endothelial Cells: The Effect of Glutaraldehyde Fixation Manifested by Characteristic Bands on the Raman Spectra of Cells. *Spectrochim. Acta - Part A Mol. Biomol. Spectrosc.* **2020**, *240*, 118460, doi:10.1016/j.saa.2020.118460.

2. Majzner, K.; Chlopicki, S.; Baranska, M. Lipid Droplets Formation in Human Endothelial Cells in Response to Polyunsaturated Fatty Acids and 1-Methyl-Nicotinamide (MNA); Confocal Raman Imaging and Fluorescence Microscopy Studies. *J. Biophotonics* **2016**, *9*, 396–405, doi:10.1002/jbio.201500134.

3. Czamara, K.; Majzner, K.; Pacia, M.Z.; Kochan, K.; Kaczor, A.; Baranska, M. Raman Spectroscopy of Lipids: A Review. *J. Raman Spectrosc.* **2015**, *46*, 4–20, doi:10.1002/jrs.4607.

4. Prescott, B.; Steinmetz, W.; Thomas, G.J. Characterization of DNA Structures by Laser Raman Spectroscopy. *Biopolymers* **1984**, *23*, 235–256, doi:10.1002/bip.360230206.

5. Wu, M.; Pu, K.; Jiang, T.; Zhai, Q.; Ma, Z.; Ma, H.; Xu, F.; Zhang, Z.; Wang, Q. Early Label-Free Analysis of Mitochondrial Redox States by Raman Spectroscopy Predicts Septic Outcomes. *J. Adv. Res.* **2021**, *28*, 209–219, doi:10.1016/j.jare.2020.06.027.

6. Bik, E.; Mielniczek, N.; Jarosz, M.; Denbigh, J.; Budzynska, R.; Baranska, M.; Majzner, K. Tunicamycin Induced Endoplasmic Reticulum Changes in Endothelial Cells Investigated: In Vitro by Confocal Raman Imaging. *Analyst* **2019**, *144*, 6561–6569, doi:10.1039/c9an01456j.

7. Matthäus, C.; Chernenko, T.; Newmark, J.A.; Warner, C.M.; Diem, M. Label-Free Detection of Mitochondrial Distribution in Cells by Nonresonant Raman Microspectroscopy. *Biophys. J.* **2007**, *93*, 668–673, doi:10.1529/biophysj.106.102061.

8. Meade, A.D.; Clarke, C.; Draux, F.; Sockalingum, G.D.; Manfait, M.; Lyng, F.M.; Byrne, H.J. Studies of Chemical Fixation Effects in Human Cell Lines Using Raman Microspectroscopy. *Anal. Bioanal. Chem.* **2010**, *396*, 1781–1791, doi:10.1007/s00216-009-3411-7.

9. Sahu, R.K.; Argov, S.; Salman, A.; Huleihel, M.; Grossman, N.; Hammody, Z.; Kapelushnik, J.; Mordechai, S. Characteristic Absorbance of Nucleic Acids in the Mid-IR Region as Possible Common Biomarkers for Diagnosis of Malignancy. *Technol. Cancer Res. Treat.* **2004**, *3*, 629–638, doi:10.1177/153303460400300613.

10. Szafraniec, E.; Wiercigroch, E.; Czamara, K.; Majzner, K.; Staniszewska-Slezak, E.; Marzec, K.M.; Malek, K.; Kaczor, A.; Baranska, M. Diversity among Endothelial Cell Lines Revealed by Raman and Fourier-Transform Infrared Spectroscopic Imaging. *Analyst* **2018**, *143*, 4323–4334, doi:10.1039/c8an00239h.

11. Perez-Guaita, D.; Kochan, K.; Martin, M.; Andrew, D.W.; Heraud, P.; Richards, J.S.; Wood, B.R. Multimodal Vibrational Imaging of Cells. *Vib. Spectrosc.* **2017**, *91*, 46–58, doi:10.1016/j.vibspec.2016.07.017.

12. Wiercigroch, E.; Staniszewska-Slezak, E.; Szkaradek, K.; Wojcik, T.; Ozaki, Y.; Baranska, M.; Malek, K. FT-IR Spectroscopic Imaging of Endothelial Cells Response to Tumor Necrosis Factor-α: To Follow Markers of Inflammation Using Standard and High-Magnification Resolution. *Anal. Chem.* **2018**, *90*, 3727–3736, doi:10.1021/acs.analchem.7b03089.

13. Banyay, M.; Sarkar, M.; Gräslund, A. A Library of IR Bands of Nucleic Acids in Solution. *Biophys. Chem.* **2003**, *104*, 477–488, doi:10.1016/S0301-4622(03)00035-8.

14. Staniszewska, E.; Malek, K.; Baranska, M. Rapid Approach to Analyze Biochemical Variation in Rat Organs by ATR FTIR Spectroscopy. *Spectrochim. Acta - Part A Mol. Biomol. Spectrosc.* **2014**, *118*, 981–986, doi:10.1016/j.saa.2013.09.131.
